# Developing a core outcome set for Menière’s disease trials, the COSMED study: a scoping review on outcomes used in existing trials

**DOI:** 10.3389/fneur.2025.1516350

**Published:** 2025-03-11

**Authors:** Maud M. E. Boreel, Babette F. van Esch, Maartje A. van Beers, Diego Kaski, Tjasse D. Bruintjes, Peter Paul G. van Benthem

**Affiliations:** ^1^Department of Otorhinolaryngology—Head and Neck Surgery, Leiden University Medical Center, Leiden, Netherlands; ^2^Apeldoorn Dizziness Centre, Gelre Hospital, Apeldoorn, Netherlands; ^3^Department of Neuro-otology, Imperial College London, London, United Kingdom

**Keywords:** Menière’s disease, randomized controlled trial, outcome domain, outcome measurement instrument, core outcome set

## Abstract

**Introduction:**

Menière’s disease (MD) is an inner ear disorder characterized by episodic vertigo, fluctuating sensorineural hearing loss, tinnitus, and aural fullness. As of yet, the etiology of MD remains unknown, which contributes to the lack of an evidence-based treatment. Outcomes and outcome measurement instruments (OMIs) used in trials assessing the effectiveness of potential MD treatment are randomly selected due to the absence of established guidelines on this matter. The objective of this review is to give an overview of the outcome domains, outcomes and OMIs used in randomized controlled trials (RCTs) evaluating treatment effects in MD to 2024. This will be the first step of developing a Core Outcome Set (COS) for MD trials.

**Methods:**

A literature search of the PubMed, Embase and Cochrane library databases was conducted from inception to November 2024. All RCTs on the treatment effect of various therapies for patients suffering from MD were included. Among other details, we extracted and analyzed all outcome domains, outcomes, and OMIs used in these RCTs.

**Results:**

A total of 76 RCTs were included, revealing a diverse range of outcomes and OMIs used across the included studies. Outcome domains encompassed dizziness, hearing, tinnitus, aural fullness, quality of life (QoL) and other. Outcomes used most frequently included: the severity of vertigo attacks, the number of vertigo attacks, vestibular function, hearing loss, severity of hearing loss, QoL related to dizziness, and Qol related to tinnitus. The latter two were most commonly measured with the Dizziness Handicap Inventory (DHI), the Functional Level Scale (FLS) and the Tinnitus Handicap Inventory (THI) respectively. For the other outcomes, there was little uniformity in the use of OMIs. Moreover, there was a notable lack of validated OMIs used in the included RCTs.

**Conclusion:**

This scoping review highlights the need for standardizing outcome selection for RCTs focusing on the treatment of MD. In this first step of developing a Core Outcome Set for MD, we identified a potential list of outcomes to be used in the next steps of ‘the Core Outcome Set for Menière’s Disease (COSMED)’ study.

## 1 Introduction

Menière’s disease (MD) is an inner ear disorder defined by intermittent spontaneous episodes of vertigo, objectified fluctuating bass-perceptive sensorineural hearing loss, tinnitus, and/or aural fullness (1). The etiology of MD is poorly understood and the definite diagnosis is made based on the above-mentioned symptoms. Partly due to its unknown etiology, an evidence-based treatment has not yet been determined (2). Clinical research on the treatment of MD is being conducted globally, but outcome measures typically differ.

In research, an outcome domain refers to a key area of interest that is relevant to a particular clinical field and can be divided in multiple outcomes. An outcome refers to what is being measured to assess the effect of an exposure or health intervention. An outcome measurement instrument (OMI) is a tool to measure that outcome. OMIs can have various forms such as a single question, a quantitative test or a questionnaire. In MD, outcomes and OMIs used in clinical trials to evaluate effectiveness are generally randomly selected due to the absence of established guidelines on this matter (1). Therefore, multiple studies have emphasized the need for identifying and standardizing relevant outcomes and OMIs used in research focused on assessing treatment options for MD (3, 4). A correct selection of outcomes and its accompanying OMIs in clinical trials is crucial because of the significant impact on patient care. Poor choice of OMIs affects the quality and clinical relevance of trial results. Moreover, inconsistency in the selection of outcomes and OMIs between trials results in failure of the synthesis of evidence and makes comparison of treatment options complicated if not impossible.

A core outcome set (COS) is a consensus-derived set for the least amount of data that ought to be measured and reported in each clinical trial for a particular condition. Implementing a COS in all trials conducted for a specific condition helps to minimize the inefficient and unethical measurement of irrelevant outcomes, thereby minimizing costs, improving quality and comparability. More importantly, the COS reflects the perspectives of various stakeholders worldwide and contains the outcomes that are most important to patients. The use of a COS enhances consistency across different trials, facilitating effective comparison and pooling of results, while also minimizing selective reporting bias (5). Due to the lack of uniformity in selecting outcomes in research focused on MD, ‘the Core Outcome Set for Menière’s Disease (COSMED)’ study aims to develop a standardized, minimal set of outcomes for randomized controlled trials on the treatment of MD.

The development of a COS requires a multi-step consensus process that includes key stakeholder groups. The Core Outcome Measures in Effectiveness Trials organization (COMET) has developed a gold-standard approach to develop a COS which consists of two stages: (1) identify outcomes that should be measured and reported and (2) assess the OMIs most appropriate to measure these outcomes.

As a first step of developing the COSMED, we have conducted a scoping review to give an overview of the outcome domains, outcomes and OMIs used in randomized controlled trials evaluating treatment effects in MD to 2024. The results of this scoping review can be used in subsequent stages of the COS development.

## 2 Methods

This scoping review was conducted in accordance with the PRISMA guidelines for scoping reviews (6). The first step in developing a COS is defining a scope. This refers to the particular area of health care of interest. The scope includes information on the target population, health condition, and interventions the COS intends to be applied to. We developed a search strategy for this scoping review based on the scope we formulated.

### 2.1 Search strategy and data sources

PubMed, Embase and Cochrane library databases were searched for eligible Randomized Controlled Trials (RCTs) on the treatment effect of various therapies for patients suffering from MD from inception to November 2024. With the assistance of a clinical librarian of the Leiden University Medical Center (LUMC), the following research strategy was comprised: (“Meniere Disease”[Majr] OR “Meniere’s”[tiab] OR “Menieres”[tiab] OR “Meniere”[tiab] OR “Ménière’s”[tiab] OR “Ménières”[tiab] OR “Ménière”[tiab] OR “Méniere’s”[tiab] OR “Ménieres”[tiab] OR “Méniere”[tiab] OR “Menière’s”[tiab] OR “Menières”[tiab] OR “Menière”[tiab] OR “Endolymphatic Hydrops”[Majr] OR “Endolymphatic Hydrops”[ti] OR ((cochlea*[ti] OR labyrinth*[ti] OR aural[ti] OR auditory[ti] OR otogenic[ti]) AND (“Vertigo”[Majr] OR “Vertigo”[ti] OR hydrops[ti] OR “Syndrome”[majr] OR syndrom*[ti]))) AND (“study”[tw] OR “studies”[tw] OR clinical trial*[tw] OR “Clinical Trial”[Publication Type] OR RCT*[tw] OR “Therapeutics”[Mesh] OR therap*[tw] OR treatment*[tw] OR intervention*[tw] OR “Treatment Outcome”[Mesh] OR outcome*[tw] OR “effectiveness”[tw] OR “efficacy”[tw] OR “end point”[tw] OR “endpoint”[tw]) NOT (Case Reports[ptyp] OR Case Report*[ti])).

### 2.2 Study selection

We included RCTs on pharmacological and non-pharmacological treatments for patients with MD. Studies with patients under the age of 18, with a sample size of less than 10, and studies that included patients with a variety of vertigo-related conditions, in which the proportion of results related to patients with MD remained unclear, were excluded. Reviews, animal studies, opinion papers and case reports were also excluded. There were no restrictions on the type of interventions or language. First, all titles and summaries were screened for potentially eligible RCTs. Secondly, articles were evaluated for eligibility by studying abstracts and full-text if necessary. Subsequently, the final decision on inclusion of the study was made.

### 2.3 Data extraction

Data was extracted using a data extraction form in Excel. For all included studies, details such as title, authors, study design, study year, country, number of participants, intervention specifics, time points and outcomes were systematically assessed and documented. The outcomes and OMIs were extracted from the method and results section of each paper. Subsequently, for each study, we categorized outcomes and OMIs into 6 different outcome domains: dizziness, hearing, tinnitus, aural fullness, quality of life and other. At the end of the data extraction process, a comprehensive list of outcomes and OMIs was conducted. This final list also specified which studies utilized which outcomes and OMIs. For each outcome domain, we provided an overview outlining the used outcomes and OMIs across the included studies.

## 3 Results

**Figure 1:**
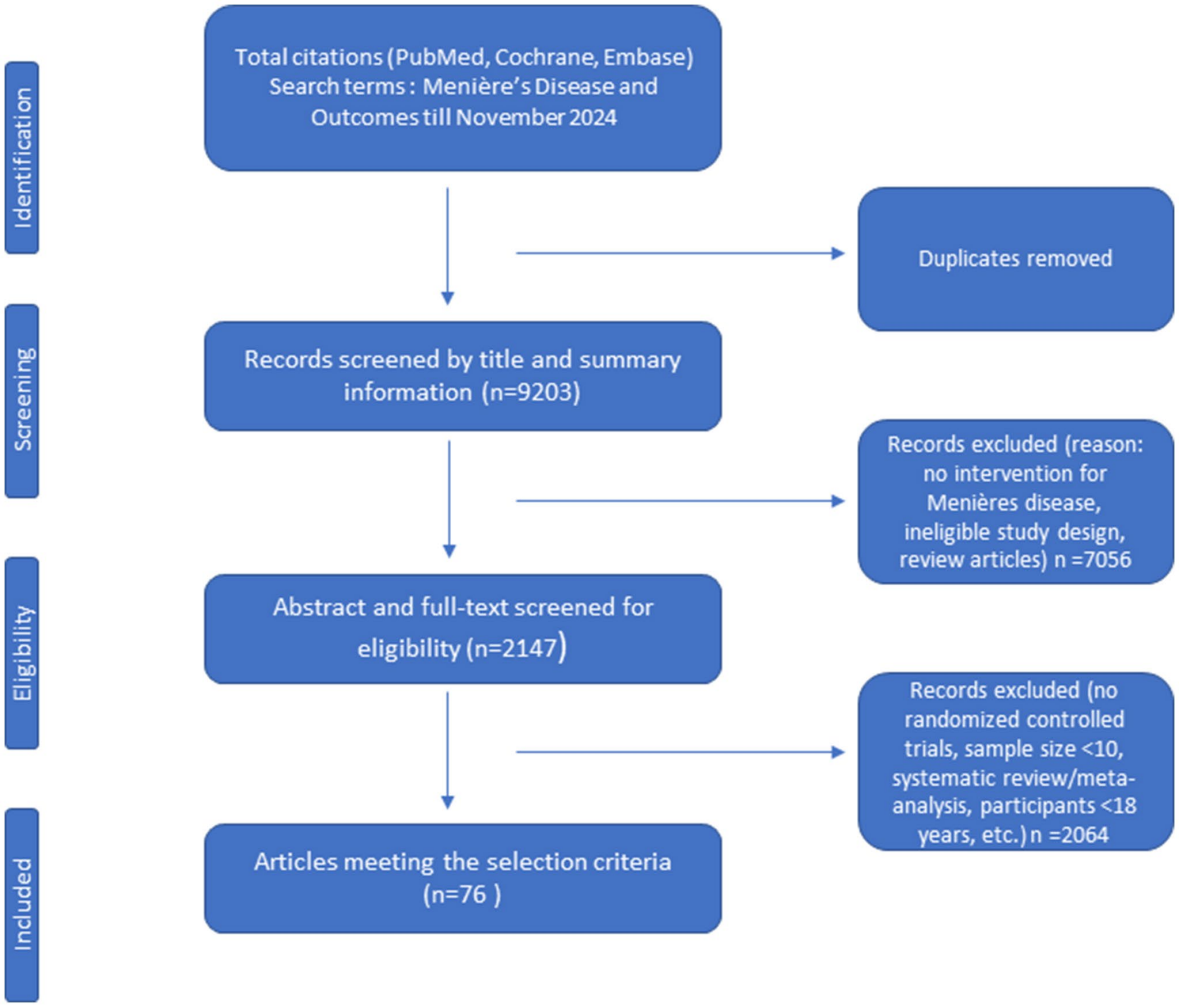
Flowchart for sorting search results and selecting studies for inclusion.

The PubMed, Embase and Cochrane library databases were searched to November 2024. In total, 9,203 studies were initially identified. Then 7,056 articles were excluded after evaluating the title and summary information. After reviewing both the abstracts and full-texts, 2,147 articles were excluded. As a result, a total of 76 RCTs were included in this study (Figure 1).

From these 76 included studies a total of 38different outcomes and 208 OMIs were identified. An overview of the included studies is presented in Table 1. An overview of all outcome domains, outcomes and OMIs used in the included studies, is presented in Supplementary Table 1.

**Table 1:** Overview of included studies.

Included study	Study design	Country of study	Intervention	Number of participants	Timepoints
Aantaa et al. (1976) (17)	Controlled clinical trial	Denmark	Betahistine hydrochloride (8 mg) vs. prochlorperazine maleate (5 mg)	30	4 and 8 months
Adrion et al. (2016) (18)	Multicenter, double-blind, randomized, placebo controlled, dose-defining trial	Germany	Placebo vs. betahistine (24mg2d) daily vs. betahistine (48mg3d)	221	1, 4, 6 and 9 months
Albu et al. (2015) (19)	Randomized controlled trial	Romania	IT dexamethasone (4 mg/ml) vs. IT dexamethasone + betahistine (48mg3d)	66	Every 2 months with a total follow-up time of 24 months
Alex et al. (2024) (20)	Randomized controlled trial	India	IT Gentamicin (40 mg/ml) vs. IT methylprednisolone (40 mg/ml) 4 injections given on alternate days.	40	3 months, a period ranging from 24 to 48 months
Bae et al. (2021) (21)	Single-institutional, single-blinded, randomized clinical trial	South-Korea	Gentamicin with normal saline vs. gentamicin with dexamethasone (no dose mentioned)	37	Every 2–3 months until 12 months after the procedure
Bojrab et al. (2018) (22)	Randomized, prospective single-blinded placebo-controlled study.	USA	Endolymphatic sac decompression + steroid injection (dexamethasone (10 g/ml) vs. endolymphatic sac decompression)	35	2, 6, 12 and 24 months
Bremer et al. (2014) (23)	Randomized, double-blind, placebo-controlled trial	Netherlands	IT gentamicin (40 mg/ml) vs. placebo	15	Every 3 months for 2 years
Burkin et al. (1967) (24)	Double-blind, randomized, cross-over study	USA	Betahistine (4mg4d) vs. placebo	22	2 and 4 weeks
Casani et al. (2011) (25)	Randomized controlled trial	Italy	IT gentamicin (40 mg/ml) vs. IT dexamethasone (4 mg/ml)	60	1 month, 1, 2 years
Covelli et al. (2017) (26)	Randomized controlled trial	Italy	1 month of self-administered low-pressure therapy + tympanostomy vs. tympanostomy only	20	30 days, 3, 6 months
Choudhary et al. (2019) (27)	Randomized controlled trial	India	Intratympanic gentamicin (20 mg/ml) vs. conservative management	32	6 months
van Deelen et al. (1986) (28)	A double-blind cross-over placebo-controlled study	Netherlands	Dyazide (50 mg triamterene and 25 mg hydrochlorothiazide) vs. placebo	33	Every 3 weeks for 34 weeks
Densert et al. (1997) (29)	Placebo-controlled, randomized clinical study	Sweden	Middle ear pressure application vs. placebo	39	Before and after exposure to treatment
Derebery et al. (2004) (30)	Randomized, double-blind, placebo-controlled clinical trial	USA	Famciclovir (250mg3d first 10 days), then (250mg2d for 80 days) vs. placebo	23	3 months
ElBeltagy et al. (2012) (93)	Comparative, prospective randomized 1-year control study	Egypt	IT dexamethasone (4 mg/ml 0.4 ml) vs. IT gentamycin (40 mg/ml 0.4 ml)	30	6 months and 1 year
Elia et al. (1964) (31)	Double-blind, cross-over evaluation	USA	Betahistine hydrochloride (16mg1d) vs. placebo	20	Every 14 days for 8 weeks
El Shafei et al. (2020) (32)	Randomized controlled trial	Egypt	IT dexamethasone (4 mg/ml) vs. IT dexamethasone (4 mg/ml) through a grommet tube vs. saline (1 ml) through grommet tube	60	3 and 6 weeks and 3, 6, 12, and 18 months
Frew et al. (1976) (33)	Double-blind, placebo-controlled cross-over trial	Netherlands	Betahistine (8mg4d) vs. placebo	28	Every 4 weeks during 36 weeks
Ganança et al. (2009) (34)	Randomized, open-label trial	Brazil	Betahistine (24mg2d) vs. betahistine (16mg3d)	120	4, 12 and 24
Garcia et al. (2013) (35)	Randomized controlled cohort study	Brazil	Virtual reality-based balance rehabilitation program vs. no program	44	6 weeks
Garduño-Anaya et al. (2005) (36)	Prospective, randomized, double-blind study	Mexico	Dexamethasone IT (4 mg/ml) vs. placebo	22	1, 6, 12,18, and 24 months
Gates et al. (2004) (37)	Randomized, double-blind, placebo-controlled, multicenter trial	USA	Active Meniett device vs. placebo (identical device that did not generate pressure)	62	Daily (vertigo) + every 3 months (questionnaires)
Guo Fang. (2007) (38)	Randomized controlled trial	China	Both groups: IV drip with 250 ml of 5% glucose, 20 ml of salvia injection and 10 mg of 654–2 at the acute stage and oral administration of sibelium (10mg1d) at the remission stage vs. intervention (acupuncture)	65	Before and after treatment
Gürkov et al. (2012) (39)	Randomized, placebo-controlled, double-blinded, clinical trial	USA	Meniett low-pressure generator vs. placebo (same device but slight pressure increase)	68	1,2,3 and 4 months
Guyot et al. (2008) (40)	Double-blind, randomized controlled trial	Switzerland	intratympanic injection with ganciclovir (50 mg/ml) vs. placebo (NaCl 9%)	29	30, 60, 90 days
Hanner et al. (2010) (41)	Prospective, double-blind, placebo-controlled study	Sweden	Specially processed cereals (1 g/kg body weight/day) vs. placebo	51	3 months
Hsu et al. (2016) (42)	Single-blind, randomized controlled trial	Taiwan	Virtual reality vestibular rehabilitation vs. Cawthorne–Cooksey vestibular rehabilitation	70	4 weeks
Khan et al. (2011) (43)	Randomized controlled trial	Pakistan	Amiloride (5mg1d) + hydrochlorothiazide (50mg1d) vs. betahistine (48mg3d) vs. multivitamin	120	1 year
Kitahara (1986) (44)	Multicenter, double-blind trial	Japan	Isosorbide (63 g) vs. betahistine mesylate (36mg3d) vs. placebo	171	2 and 4 weeks
Kitahara et al. (2008) (45)	Randomized controlled trial	Japan	Endolymphatic sac drainage + steroid instillation vs. endolymphatic sac drainage without steroid instillation vs. control (declined endolymphatic sac drainage)	197	At least 2 years
Kitahara et al. (2016) (46)	Randomized controlled and open-label trial	Japan	Traditional oral medication (diuretics, betahistine, diphenidol, dimenhydrinate and diazepam) vs. abundant water (35 ml/kg/day) intake + traditional oral medication vs. tympanic ventilation tubes + traditional oral medication vs. sleeping in darkness + traditional oral medication.	297	Once a month for at least 24
Lambert et al. (2012) (47)	Double-blind, randomized, placebo-controlled, dose-escalation study	USA	IT OTO-104 (3 mg) vs. IT OTO-104 (12 mg) vs. placebo	44	3 months
Lambert et al. (2016) (48)	Randomized controlled trial	USA	IT Oto-104 (60 mg/ml) vs. placebo	154	1, 2, 3, 4 months
Leong et al. (2013) (49)	Randomized double-blinded, placebo-controlled, crossover study	England	Specially processed cereal vs. placebo	39	3 months
Liu et al. (2020) (50)	Randomized controlled trial	China	Betahistine (12mg3d) vs. vestibular rehabilitation (Tetrax biofeedback) vs. no treatment	66	1 month
Lyu et al. (2020) (51)	Randomized controlled trial	China	Post-operative IV dexamethasone (10mg1d) vs. post-operative no dexamethasone	124	1 week, 1, 6 months and 2 years
Martini et al. (1990) (52)	Randomized, double-blind study	Italy	Trimetazidine (60 mg/d) vs. betahistine (12mg3d)	45	1 and 6 months
Masumi et al. (2017) (53)	Randomized controlled trial	Iran	IT dexamethasone (4 mg/dL) vs. IT methylprednisolone (40 mg/dL)	69	1 and 6 months
Meyer et al. (1985) (54)	Double-blind, crossover trial vs. placebo	Germany	Betahistine (36 mg) vs. placebo	40	2, 6, 12 weeks and 1 year
Mira et al. (2003) (55)	Double-blind, multi center and parallel-group randomized trial	Italy	Betahistine (16mg2d) vs. placebo	144 (81 with Menière’s Disease)	15, 30, 60 and 90 days
Miura et al (1994) (56)	Randomized controlled study	Japan	Ibudilast (10mg3d) vs. nothing	46	After treatment (12 weeks)
Morales-Luckie et al (2005) (57)	Blinded, randomized controlled trial	Mexico	Diphenidol (25mg1d) + acetazolamide (250mg48h) vs. diphenidol (25mg1d) + acetazolamide (250mg48h) + prednisone (0.35 mg/kg/d)	16	Every 6 weeks for 1 year
Moser et al. (1984) (58)	Prospective, double-blind, cross-over, placebo-controlled trial	Austria	O-(B-hydroxyethyl)-rutosides (500mg2d) vs. placebo	39	Every month for 7 months
Novotý et al. (2002) (59)	Randomized, double-blind, reference-controlled design	Czech republic	Cinnarizine (20mg1d) + dimenhydrinate (40mg1d) vs. betahistine dimesylate (12mg3d)	82	1,3,6, and 12 weeks
Paragache et al. (2005) (60)	Prospective randomized study	India	IT dexamethasone (0.20 mg/cc) vs. nothing	40	1, 2, 3 and 6 months
Park et al. (2016) (61)	Multicenter randomized study	South Korea	Betahistine (6mg3d) vs. isosorbide (30ml3d) + betahistine (6mg3d)	220	4 and 12 weeks
Patel et al. (2016) (62)	Double-blind, randomized controlled trial	UK	IT gentamicin (40 mg/ml) vs. IT methylprednisolone (62.5 mg/ml)	60	1, 2, 6, 12, 18, and 24 months
Phillips et al. (2023) (63)	3 randomized, double-blind, placebo-controlled, multicenter phase 3 studies	USA and Europe	IT oto-104 (12 mg) vs. placebo	487	1,2 and 3 months
Postema et al. (2008) (64)	prospective, double-blind, randomized, placebo-controlled trial	Netherlands	Gentamicin sulfate (0.4 ml 30 mg/ml) vs. placebo	35	6 weeks, 6 and 12 months
Ödkvist et al. (2000) (65)	Clinical multicentre placebo-controlled study	Sweden	Overpressure treatment vs. placebo	56	2 weeks
Okamoto (1968) (66)	Randomized controlled trial	Japan	Betahistine dihydrochloride (16mg3d) vs. placebo	40	2 weeks
Rask-Andersen et al. (2005) (67)	Randomized, placebo-controlled, double-blind, pilot study	Sweden	IT Preservative-free latano-prost (50 g/ml) vs. placebo	10	5 and 15 days
Redon et al. (2011) (68)	Randomized, double-blind, placebo-controlled trial	France	Placebo vs. betahistine (24mg2d)	16	7, 30, 90 days
Ricci et al. (1987) (69)	Randomized controlled trial	Italy	Betahistine hydrochloride (8mg3d) vs. placebo	10	Every week until the end of the trial
Rizk et al. (2024) (70)	Randomized, double-blind, placebo-controlled, crossover pilot study	USA	Venlafaxine (37.5mg1d) vs. placebo	40	60 days
Russo et al. (2016) (71)	Randomized, double-blind, placebo-controlled multicenter trial	France	Meniett devise vs. placebo	199	8, 14, 20 weeks
Salami et al. (1984) (72)	Double-blind, randomized study	Italy	Betahistine hydrochloride (8mg3d) vs. placebo	30	Weekly for 6 weeks
Saliba et al. (2015) (73)	Randomized controlled trial	Canada	Endolymphatic duct blockage vs. endolymphatic sac decompression	57	1 week, 1, 6, 12, 18, 24 months
Sarafraz et al. (2015) (74)	Randomized controlled trial	Iran	IT gentamicin (27 mg/ml) vs. IT methylprednisolone (?)	20	3 months
Schmidt et al. (1992) (75)	Randomized controlled trial	Netherlands	Betahistine dihydrochloride (24mg3d) vs. placebo	40	Every month for 33 months
Scott et al. (1994) (76)	Experimental between-group, cross-over design with randomization	Sweden	First transcutaneous nerve stimulation, then applied relaxation vs. first applied relaxation, then transcutaneous nerve stimulation	20	4, 6, 9 and 16 weeks.
Silverstein et al. (1998) (77)	Prospective, randomized, double-blind, crossover study	USA	IT dexamethasone (16 mg/ml) + sodium hyaluronate (8 mg/ml) vs. placebo	20	Every week for 6 weeks
Stokroos et al. (2004) (78)	Prospective, double-blind, Placebo-controlled, Randomized Clinical Trial	Netherlands	Gentamicin (30 mg/m) vs. placebo	22	6 months
Storper et al. (1998) (79)	Randomized, prospective study	USA	Glycopyrrolate (2 mg) vs. placebo	37	4–6 weeks
Teggi et al. (2008) (80)	Randomized pilot study	Italy	Low-level laser therapy 20 min a day with a 5-mW soft laser vs. betahistine (16mg2d)	20	3, 6 months
Thomas et al. (2021) (81)	Randomized controlled trial	India	IT buffered gentamicin (40 mg/ml) vs. IT methylprednisolone (40 mg/ml)	22	3, 24–48 months
Thomsen et al. (1981) (82)	Randomized, placebo- controlled study	Denmark	Endolymphatic sac surgery vs. sham surgery	30	Every month for 12 months
Thomsen et al. (1998) (83)	Prospective, randomized, controlled study	Denmark	Ventilation tube vs. endolymphatic sac shunt operation	29	Every month for 12 months
Thomsen et al. (2005) (84)	Clinical, randomized, multicenter, double-blind, placebo-controlled study	Denmark	Local overpressure therapy vs. placebo	40	2, 4, 8 weeks
Wilmot et al. (1976) (85)	Double-blind, placebo-controlled, cross-over, clinical study	Ireland	Betahistine (16 mg3d) vs. placebo	24	8, 12, 16 and 24 weeks
Wu et al. (2018) (86)	Randomized controlled trial	China	Both: mecobalamine (0.5mg3d) + betahistine (12 m 3d) vs. treatment (acupuncture)	96	12 weeks, 18–24 months
Wu et al. (2023) (87)	Single-blind randomized controlled trial	China	Transcutaneous auricular vagus nerve stimulation vs. sham transcutaneous auricular nerve stimulation. Both groups: betahistine (6mg3d)	104	12 weeks
Yang et al. (2024) (88)	Randomized controlled trial	China	Low-sodium diet (restricted to 1,500 mg/day) + adequate water intake (35 ml/kg/day) + routine medication vs. routine medication	50	3 months
Yardley et al. (2006) (89)	Randomized controlled trial	UK	Vestibular rehabilitation booklet vs. symptom control booklet vs. waiting list	360	3, 6 months
Yin et al. (2022) (90)	Randomized controlled trial	China	IT dexamethasone ITD1 (2 mg/ml) and ITD2 (5 mg/ml) vs. IT lidocaine (20 mg/ml)	124	1 month
Zarandi et al. (2023) (91)	Randomized controlled Trial	Iran	*Nigella sativa* oil (1 g capsules) vs. placebo	40	3 months
Zhuang et al. (2022) (92)	Randomized controlled trial	China	Routine outpatient treatment + vestibular rehabilitation for 8 weeks vs. routine outpatient treatment	45	8 weeks

### 3.1 Dizziness

The first and most frequently reported outcome domain in all studies was dizziness. Within this outcome domain, we extracted several outcomes including the number of vertigo attacks, vertigo severity, duration of a vertigo attack, type of episode and vertigo control/improvement. A wide variety of OMIs were used to evaluate these outcomes. Most of these OMIs were non-validated, self-created, symptom assessments or rating scales. The OMI that was used most frequently was the ‘class of vertigo control’, defined according to the AAO-HNS criteria (1). Additionally, within this outcome domain, a wide range of rating scales have been used to measure vertigo severity and vertigo control. Various questionnaires including the Dizziness Handicap Inventory (DHI) (7) and the Functional Level Score (1) were commonly used, however, these instruments assess vertigo-related quality of life (QoL) and thus are categorized under the outcome domain Qol.

### 3.2 Hearing

Given that hearing loss, similar to dizziness, serves as a diagnostic prerequisite for MD, numerous studies included hearing loss as an outcome. However, little uniformity was found in the frequencies of pure tone average (PTA) to be measured, with some studies not even specifying the frequencies assessed. In line with the AAO-HNS criteria (1), frequencies most measured were 0.5, 1, 2 and 3 kHz. Seventeen studies measured word recognition scores in addition to PTA (WRS). To determine the statistical significance of hearing loss, most studies assessing hearing, used the AAO-HNS scale where a change of more than 10 decibels in PTA, or a change of 15% in WRS was defined as a clinically significant difference (1).

### 3.3 Tinnitus

Articles including tinnitus as an outcome frequently faced challenges in specifying their measurement methods. Frequency of tinnitus, tinnitus prevalence, tinnitus presence or absence and the degree of tinnitus improvement were terms that were used in the articles but were not explicitly defined. Eleven different likert scales were used to measure tinnitus severity by 19 different studies. The Tinnitus Handicap Inventory (THI) (8) was most commonly used, but, similar to the DHI, we categorized this under the core domain QoL.

### 3.4 Aural fullness

Only 22 out of the 71 RCTs included, measured aural fullness as an outcome measure. All of these studies used OMIs that were not validated such as a variety of different likert scales, recorded in a diary or presence or absence. Furthermore, several studies did not provide details regarding the method used to measure aural fullness.

### 3.5 Quality of life

The assessment of QoL was categorized into several outcome domains, including QoL related to dizziness and tinnitus. Among the included RCTs, for the evaluation of QoL the Dizziness Handicap Inventory (DHI) (7), Tinnitus Handicap Inventory (THI) (8), and the Functional Level Scale (FLS) defined by the AAO-HNS criteria (1), were predominantly used. Additionally, a variety of questionnaires were used to measure quality of life in relation to dizziness, including the European Evaluation of Vertigo (EEV) scale (9), Vertigo Symptom Scale short form (VSS-SF) (10), Menière’s Disease Patient-Oriented Symptom Severity Index (MD-POSI) (11), Dizziness Beliefs Questionnaire (DBQ) (12), Vestibular Disorders Activities of Daily Living (VDADL) score (13), the Menière’s Disease Outcomes Questionnaire (MDOQ) (14) and the Neuropsychological Vertigo Inventory (NVI) (15). Furthermore, some studies addressed general health-related QoL or aspects related to psychological health, although these aspects were explored in a limited number of studies.

### 3.6 Other

The most frequently used outcomes which were categorized under ‘other’ were vestibular function, auditory function and balance. The most commonly utilized clinical tests were electronystagmography and electrocochleography. Balance was assessed using a wide range of clinical tests and rating scales.

Other outcome measurements that were evaluated included compliance, tolerability and overall judgment of treatment, quality of appointments, laboratory tests, activity level and other general symptoms, possibly related to MD. These outcome measurements were primarily related to interventions and were highly specific for the respective studies.

### 3.7 Adverse events

Among all studies included, a wide variety of adverse events was reported. Mostly, the adverse events reported were depending on the specific intervention that was performed and reflecting the risks associated with each intervention type. Due to this wide variation, which was often linked to the type of intervention, we decided not to include adverse events in the list of outcomes for MD.

## 4 Discussion

The key finding of this scoping review was the significant diversity observed in outcome domains, outcomes and OMIs reported across all RCTs included. The outcome domain dizziness was most commonly used and used in 64 out of the 76 studies. Eight additional studies exclusively used the DHI or the VSS-sf to evaluate dizziness, which were categorized under QoL related to dizziness. In total, four articles did not assess dizziness in any capacity. Hearing and tinnitus, which were the next most used domains, and were reported in 61 and 33 studies respectively, whereas 16 studies reported the quality of life related to tinnitus using the THI. Quality of life (QoL) was assessed in 35 studies, with a trend indicating that QoL was evaluated less frequently in the older studies. Aural fullness was the least reported outcome domain, featuring in only 22 studies. A notable issue within the outcome domains aural fullness and tinnitus was the lack of clarity regarding the assessment method used to measure outcomes. For instance, some studies evaluated tinnitus loudness or frequency without specifying the measurement method. We concede that tinnitus and aural fullness are challenging symptoms to classify, however, the lack of clarity on how outcome domains were assessed raises concerns about the reliability of these findings. The vestibular function, auditory function and balance categorized under the outcome domain ‘other’ were measured in 34 studies where in the vast majority of studies electronystagmography or electrocochleography was performed.

There was no single outcome that was reported in all studies. However, in the outcome domain QoL, there was a considerable overlap in the OMIs used. QoL related to dizziness was assessed using the DHI in 21 studies and using the FLS in 13 studies. Similarly, QoL related to tinnitus was measured using the THI in 16 studies. The OMI electronystagmography (caloric test) was the OMI most frequently used and was measured in 19 studies. In contrast, there was not much similarity in the OMIs used to measure the outcomes in the other domains. The ‘severity of vertigo attacks’ was the most commonly utilized outcome, though it was assessed using 24 distinct outcome measurement instruments (OMIs). Additionally, the outcome measure number of vertigo attacks was measured in as many as 35 studies using 17 different OMIs. Similarly for the outcome hearing loss, where various combinations of frequencies were used to calculate the PTA. The variation in OMIs used to measure a specific outcome, complicates the comparison of findings between studies. For example, when measuring vertigo severity, studies may use patient self-reported scales, frequency counts of vertigo attacks, or standardized questionnaires like the DHI. A study focusing on attack frequency alone may show different treatment effects than one measuring intensity or overall handicap. This variability makes it challenging to interpret which treatments are genuinely effective across different vertigo dimensions. Such inconsistencies also complicate the development of clinical recommendations, as different aspects of vertigo may influence treatment guidance differently.

Alongside the variation in the use of OMIs, there was an evident absence of validated measurement instruments. For example, to measure vertigo severity, many different rating scales were used. To our knowledge, these scales are not validated.Without proper validation, it is uncertain whether the instrument captures the relevant aspects of the outcome and if it is sensitive enough to detect meaningful changes and which cut-off point are defined as ‘clinically relevant’. This could lead to a misinterpretation of the true effect of the intervention. Furthermore validation establishes that an OMI can be reliably applied across different populations and settings. Non-validated instruments may perform inconsistently across diverse demographic or clinical groups, limiting the comparability of findings. In addition, we could not find any validation studies on the OMIs proposed by the AAO-HNS such as the Functional level score, the class of vertigo control, or the hearing severity scale. However, even though validated questionnaires, like the DHI, are commonly used, their quality may not always be optimal. In a study by Koppelaar-van Eijsden et al. (16), the quality of DHI was assessed using the COnsensus-based Standards for the selection of health Measurement INstruments (COSMIN) methodology. They concluded that the current evidence for several measurement properties is suboptimal. Furthermore, the DHI might not be the best tool measure vertigo in MD specifically. Furthermore, the DHI may not be the most suitable instrument for assessing vertigo in MD specifically. Firstly, it is not well-suited for episodic syndromes, particularly those characterized by fluctuating symptom severity or clustered attacks. Additionally, the term ‘dizziness’ may not accurately capture the experiences of many MD patients, who often suffer from severe vertigo rather than generalized dizziness.

One of the key strengths of this scoping review is that we did not exclude any studies based on language or availability. In case studies were not available in full text through our institution, we purchased them, if possible, online. Four studies were not available in English and were translated with the aid of artificial intelligence tools. This comprehensive approach ensures that we have captured all outcomes used in recent RCTs in this study.

A limitation of this scoping review was the variability in outcome measures and OMIs across studies, which made data categorization challenging. Several OMIs did not fit into any specific domain, resulting in their classification under the domain “other,” which may reduce the clarity of the overview. Another limitation of this study is that we only assessed studies with two reviewers when there was uncertainty about their inclusion. This may affected the reliability of the selection process.

As previously mentioned, this review is part of the COSMED-study, which will be conducted in two distinct stages. The first stage will focus on identifying outcome domains for inclusion in the COS while the second stage will determine the specific outcomes to assess these domains. To reach consensus on the outcome domains and outcomes to be incorporated into the COS, a Delphi procedure will be conducted at each stage, engaging experts on MD to give their valuable opinion.

This review has collected an extensive list of all outcome domains, outcomes, and OMIs reported in recent RCTs on the treatment of MD and will serve as a basis for the upcoming Delphi procedures. However, it is important to acknowledge that this list primarily reflects the perspectives of clinicians and researchers, potentially overlooking the valuable input of patients. Patients often provide unique perspectives on disease symptoms and how these symptoms affect their daily lives, which may differ significantly from the view of clinicians and researchers. Thus, patient involvement is critical in selecting the outcomes and OMIs to be included in the COS.

To integrate the patient perspective, a focus group meeting with patients will be conducted. In this initial meeting, we will discuss outcome domains that are particularly meaningful for patients with MD. In a subsequent patient focus group during the second stage of the study, we will focus specifically on identifying outcomes that are highly relevant to patients. These patient-derived domains and outcomes, together with those identified through the review, will form the basis of the Delphi procedures for the COSMED-study.

In conclusion, there is a lack of standardized outcomes and OMIs used in RCTs on the treatment of MD. To address this problem, the COSMED study aims to develop a COS for use in RCTs evaluating MD treatments. As a first step, in this study we identified outcome domains, outcomes, and OMIs used in RCTs on the treatment of MD to 2024. The identified outcome domains encompass dizziness, hearing, tinnitus, aural fullness, Qol and other, with a notable variability in outcomes and OMIs observed within these outcome domains. Additionally, we found a lack in the use of validated OMIs. Together with the inconsistency in outcomes and OMIs reported, we emphasize the need for a standardized COS for MD. In this first step of developing a COS for MD, we identified a potential list of outcomes to be used in the next steps of our COSMED study. The development of a COS holds promise for enhancing consistency, comparability, and relevance in evaluating treatment outcomes for MD, ultimately improving patient care and advancing clinical research in this field.
