# Supplementary material for: Developing a core outcome set for Menière’s disease trials, the COSMED study: a scoping review on outcomes used in existing trials
Source: Front Neurol. 2025 Mar 11;16:1516350. doi: 10.3389/fneur.2025.1516350 (PMC11956534; doi:10.3389/fneur.2025.1516350)
Supplement: Supplementary file 1 [file Table_1.pdf]

Table 1. **Overview of outcome domains, outcomes and outcome measurement instruments used in the included studies. AAO-HNS, American Academy of Otolaryngology–Head and Neck Surgery; PTA, Pure Tone Average.**

Outcome domain	*Outcomes*	Outcome measurement instrument (OMI)	Studies
**Vertigo**	*Number of Vertigo attacks*	Number of definite vertigo days	Gürkov et al. (2012), Lambert et al. (2016)
		Number of definitive vertigo days per month	Lambert et al. (2012), Phillips et al. (2023)
		Number of vertigo attacks per week	Salami et al. (1984)
		Number of vertigo attacks per month	Bojrab et al. (2018), Garduño-Anaya et al. (2005), Mira et al. (2003), Ricci et al. (1987), Schmidt et al. (1992)
		Number of vertigo attacks per study phase	Rizk et al. (2024), Russo et al. (2016)
		Number of vertigo attacks every follow-up year compared to the 6 months before intervention	Kitahara et al. (2008)
		Number of vertigo attacks after 24 months of follow-up compared to 6 months before the intervention	Saliba et al. (2015)
		Number of vertigo attacks 18-24 months after intervention compared with the 6 months before intervention	Kitahara et al. (2016), Patel et al. (2016)
		Number of vertigo attacks 18-24 months after intervention compared with the 1 month before intervention	Patel et al. (2016)
		Number of vertigo attacks in the 6 months after intervention compared with 6 months before the intervention	Choudhary et al. (2019)
		Number of vertigo attacks in the 4 months after intervention compared with 4 months before the intervention	Park et al. (2016)
		Mean vertigo attacks per day	Lambert et al. (2016), Morales-Luckie et al (2005)
		Mean vertigo attacks per month; pre-treatment vs 3 months, > 24 months	Thomas et al. (2021)
		Change from baseline in vertigo rate	Bae et al. (2021), Lambert et al. (2012)
		Individual attack rate standardized on a 30 day interval	Adrion et al. (2016)
		Dizziness – present or absent	Burkin et al. (1976)
		Dizziness frequency (unspecified)	Aantaa et al. (1976), Alex et al. (2024), Kitahara (1986), Martini et al. (1990), Meyer et al. (1985), Scott et al. (1994), Stokroos et al. (2004), Thomsen et al. (1981), Thomsen et al. (2005)
	Vertigo severity	6-point likert scale: Vertigo score	van Deelen et al. (1986) 34
		6-point likert scale: 0 = absent, 5 = disabling (Intensity of symptoms)	Martini et al. (1990)
		5-point Likert scale: 0 = vertigo-free day, 1 = vertigo episode lasting <20 minutes, 2 = definitive vertigo episode lasting >20 minutes, 3 = definitive vertigo episode associated with nausea/vomiting, 4 = worst attack experienced to date	Gates et al. (2004), Gürkov et al (2012), Lambert et al. (2012), Lambert et al. (2016)
		5-point likert scale 0 = no symptoms and 4 = very strong symptoms (Intensity of symptoms)	Novotný et al. (2002), Wilmot et al. (1976)
		4-point likert scale: level 1 = acute vertigo occurring > 3 times and lasting > 2 h, severe nausea and vomiting, inability to walk, hospitalization needed for hydration, level 2 = acute vertigo occurring twice and lasting 1-2 h, nausea and a few vomiting episodes, inability to walk, no hospitalization, level 3 = acute vertigo occurring once and lasting less than 1 h, nausea without vomiting, ability to	Ganança et al. (2009)

	walk with assistance, no hospitalization, Level 4= no acute vertigo	
	4-point likert scale: 0 = none, 1- mild, 2 = moderate, 3 = severe,	Postema et al. (2008), Kitahara (1986), Salami et al. (1984), Schmidt (1992)
	4-point likert scale: 0 = no disability, 1 = mild disability: mild unsteadiness or dizziness that precludes working in a hazardous environment, 2 = moderate disability: moderate unsteadiness or dizziness that results in necessity for a sedentary occupation, 3 = severe disability: symptoms exclude gainful employment	Morales-Luckie et al (2005)
	4-point likert scale: 0 = absent, 1 = mild, 2= severe, 3 = very severe	Moser et al. (1984)
	4-point likert scale: 0 = not present, 1 = present but moderate, 2 = severe but not incapacitating 3 = incapacitating	Elia et al. (1964)
	4-point likert scale: 0 = no symptom at all and 4 = all 12 symptoms present with maximum intensity (dystasia and walking unsteadiness, staggering, rotary sensation, tendency to fall, lift sensation, and blackout)	Novotný et al. (2002)
	4-point likert scale: 0 = no symptom at all and 4 = all 12 symptoms present with maximum intensity (vertigo trigger factors: change of position, bowing, getting up, driving by car or train, head movements, and eye movements)	Novotný et al. (2002)
	4-point likert scale (unspecified)	Frew et al. (1976)
	3-point likert scale: 0 = no dizziness, 1 = dizziness present but does not interfere with work, 2 = dizziness severe enough to prevent working, 3 = very severe dizziness, rendering the patient unable to perform any activities	Miura et al. (1994)
	3-point likert scale: mild = vertigo with no nausea; could perform routine work, moderate = vertigo with nausea; had to suspend work during the attack, severe = vertigo + nausea + vomiting + had to stay in bed.	Khan et al. (2011)
	3-point likert scale: mild, moderate severe, very severe	Adrion et al. (2016)
	3-point likert scale: 0 = none, 1 = little bit, 2 = extreme	Okamoto et al. (1968)
	3-point likert scale (unspecified)	Thomsen et al. (1981)
	Dizziness analog scale: 0 = no vertigo to 10 = maximal intensity	Garcia et al. (2013), Guyot et al. (2008), Ödkvist et al. (2000), Rask-Andersen et al. (2005)
	GISFaV self-rating scale	Mira et al. (2003)
	Visual Vertigo Analog Scale (VVAS)	Russo et al. (2016), Thomsen et al. (2005)
	Dizziness Assessment Rating Scale (DARS)	Mira et al. (2003)
	Vertigo visual scale (unspecified)	Densert et al. (1997)
	The effect of vertigo on daily activity (days at home sick or bedridden)	Phillips et al. (2023)
	Vertigo severity (unspecified)	Aantaa et al. (1976), Phillips et al. (2023), Rizk et al. (2024)
Duration of vertigo attack	Median duration of attack: 2 = (1-20 min), 3 = (20-60 min), 4 = (60-180 min), 5 = (>180 min)	Adrion et al. (2016)
	Duration of attacks expressed in minutes	ElBeltagy et al. (2012), Teggi et al. (2008)
	Mean duration of episodes	Salami et al. (1984), Ricci et al. (1987)
	Interval between episodes	Salami et al. (1984), Ricci et al. (1987)

		Duration of vertigo attack (unspecified)	Kitahara (1986), Martini et al. (1990), Morales-Luckie et al (2005), Rizk et al. (2024), Thomsen et al. (1981)
	Type of episode	Subjective/objective	Salami et al. (1984)
		Rotational/non-rotational	Kitahara et al. (1986)
		Unspecified	Ricci et al. (1987)
	Vertigo control/improvement	Class and vertigo control (AAO-HNS criteria): A = 0, B = 1-41, C = 41-80, D = 81-120, E > 120, F = secondary treatment initiated because of disability from vertigo	Albu et al. (2015), Alex et al. (2024), Casani et al. (2011), Choudhary et al. (2019), Covelli et al. (2017), ElBeltagy et al. (2012), El Shafei et al. (2020), Morales-Luckie et al. (2005), Rizk et al. (2024), Sarafraz et al. (2015), Teggi et al. (2008), Thomsen et al. (1998), Wu. et al. (2018)
		Vertigo numeric scale (AAO-HNS criteria) A = 0-40 = complete control of definitive spells , B = 41-80 = limited control of definitive spells, C = 81-120 = insignificant control of definitive spells	Masoumi et al. (2017)
		Sakata's criteria: excellent = no effect, good = improvement of spells, unchanged= no effect, worse = increase in the attacks	Paragache et al. (2005)
		10-point likert scale: subjective vertigo improvement scale 0 = no change, 10 = 100% subjective improvement	Garduño Anaya et al. (2005)
		3-point likert scale: subjective change: 1 = improved, 2 = worse, 3 = no change	Leong et al. (2013), Meyer et al (1985)
		3 point likert scale: subjective change: 1 = modest, 2= moderate, 3 = substantial	Ricci et al. (1987)
		Complete vertigo control	Bae et al. (2021), Kitahara et a.l (2008), Kitahara et al (2016),
		Number of vertigo-free days	Gürkov et al. (2012)
		Reduction in the number of disabling vertigo episodes	Derebery et al. (2004)
		Improvement of vertigo attacks (not further specified)	Khan et al. (2011)
**Hearing**	*Hearing loss*	3 frequency average PTA: 0,25 0.5, and 1kHz	Gürkov et al. (2012), Thomsen et al. (1981)
		3 frequency average PTA: 0,5, 1 and 2 kHz	Gates et al. (2004), Kitahara et al. (2008), Lambert et al. (2016), Ödkvist et al. (2000), Ricci et al. (1987), Yang et al. (2024)
		4 frequency average PTA: 0.5, 1, 2, and 3 kHz (defined according to the AAO-HNS criteria)	Albu et al. (2015), Alex et al. (2024), Bae et al. (2021), Casani et al. (2011), Derebery et al. (2004), Lyu et al. (2020), Morales-Luckie et al (2005), Masoumi et al. (2017), Patel et al. (2016),Park et al. (2016), Raks-Andersen et al. (2005), Scott et al. (1994), Silverstein et al. (1998), Teggi et al. (2008), Thomas et al. (2021)
		4 frequency average PTA: 0.5, 1, 2, and 4 kHz	Bojrab et al. (2018), Bremer et al. (2014), Covelli et al. (2017), El Shafei et al. (2020), Kitahara et al. (2016), Meyer et al. (1985), Stokroos et al. (2004), Thomsen et al. (1998), Zarandi et al. (2023)

	4 frequency average PTA: 0.25, 0.5, 1 and 2 kHz	Adrion et al. (2016), van Deelen et al. (1986), Miura et al. (1994), Novotný et al. (2002), Salami et al. (1984), Schmidt et al. (1992)
	5 frequency average PTA: 0.25, 0.5, 1, 2, and 4 kHz	ElBeltagy et al. (2012), Saliba et al. (2015)
	5 frequency average PTA: 0.25, 0.5, 1, 2, and 3 kHz	Moser et al. (1984)
	6 frequency average PTA: 0.25, 0.5, 1, 2, 4 and 8 kHz	Wu et al. (2018), Wu et al. (2023)
	PTA high: average threshold shift of 4 and 8kHz	Bae et al. (2021)
	Pure Tone Audiometry (unspecified)	Choudhary et al. (2019), Densert et al. (1997), Frew et al. (1976), Garduño Anaya et al. (2005), Kitahara (1986), Lambert et al. (2012), Okamoto (1968), Postema et al. (2008), Thomsen (2005), Sarafraz et al. (2015), Storper et al. (1998)
	Speech audiometry (Word Recognition Score)	Albu et al. (2015), Alex et al. (2024), Casani et al. (2011), van Deelen et al. (1986), Densert et al. (1997), Derebery et al. (2004), ElBeltagy et al. (2012), Garduño-Anaya et al. (2005), Lambert et al (2012), Lyu et al. (2020), Morales-Luckie et al (2005), Ödkvist et al. (2000), Patel et al. (2016), Raks-Andersen et al. (2005), Saliba et al. (2015), Storper et al. (1998), Thomsen et al. (1998)
	Tympanometry	Lambert et al. (2012), Storper et al. (1998)
	Hearing loss (unspecified)	Paragache et al. (2005), Rizk et al. (2024)
Severity of hearing loss	AAO-HNS scale: unchanged - improved – worsened clinically significant: change of 10 dB or more in PTA	Albu et al. (2015), Casani et al. (2011), Garduño Anaya et al. (2005), Kitahara et al. (2008), Kitahara et al. (2016), Khan et al. (2011), Morales-Luckie et al. (2005), El Shafei et al. (2020), Saliba et al. (2015)
	AAO-HNS scale: unchanged – improved – worsened: clinically significant: change of 15% or more in word recognition score	Albu et al. (2015), Casani et al. (2011), Garduño Anaya et al. (2005), Morales-Luckie et al (2005), Saliba et al (2015)
	AAO-HNS hearing stage: AAO-HNS criteria: class I (<25 dB), II (26–50 dB), III (51–70 dB), and IV (>71 dB).	Bae et al. (2021), Covelli et al (2017), Kitahara et al. (2008), Masumi et al. (2017)
	>10 dB improvement = significant improvement, 5-10 dB improvement = moderate improvement, <5 dB improvement or no change = no effect	Miura et al. (1994)
	A = subjective improvement and hearing recovery >30dB, B = Hearing recovery between 15-30db, C = stability, D = worsening greater than 15db	Martini et al. (1990)
	An increase ≥10dB = hearing worsening, an increase ≥20dB = profound hearing worsening	Lyu et al. (2020)
	Grade A: improved > 30db or improved by < 20	Yang et al. (2024)

		dbHL at each frequency, grade B: improved by 15-30db, grade C: improved by 0-14db, grade D: improved by <0db.	
		A decrease ≥15% = WRS loss, a decrease ≥25% = profound WRS loss	Lyu et al. (2020)
		A change of 10db = treatment effect	Meyer et al. (1985)
		10-point likert scale (Visual Analog scale): 0 = no symptoms, 10 = maximum symptoms	Rask-Andersen et al. (2005)
		10-point likert scale: maximal ability to hear	Scott et al. (1994)
		5-point rating scale 0 = absent, 5 = disabling	Martini et al. (1990)
		4-point likert scale	Frew et al. (1976), Wilmot et al. (1976)
		4-point likert scale: 3 = severe, 2 = moderate, 1 = mild, 0 = none	Kitahara et al. (1986), Postema et al. (2008), Moser et al. (1984)
		4-point likert scale: good, slightly impaired, moderately impaired, seriously impaired	Schmidt et al. (1992)
		3-point likert scale: 0 = none, 1 = little bit, 2 = extreme	Okamoto et al. (1968)
		3-point likert scale (unspecified)	Thomsen et al. (1981)
		Goodman scale: 0-25db = normal, 26-40db = mild hearing loss, 41-55db = moderate haring loss, 56-70db = moderately serious hearing loss, 71-90db = serious hearling loss, 91db = very serious hearing loss	Salami et al. (1984), Ricci et al. (1987)
	Subjective improvement of hearing	Subjective improvement confirmed on PTA: same as before – worse – better (change > 10db)	Khan et al. (2011), Silverstein et al. (1998)
		Patient perception of hearing (VAS scale)	Thomsen et al. (2005)
		4-point likert scale: better, same, worse, fluctuating	Schmidt et al. (1992)
		10-point likert scale: subjective improvement: 0 = no change, 10 = 100% subjective improvement	Garduño Anaya et al. (2005)
	Disability due to hearing	Hearing disability scale	Yardley et al. (2006)
**Tinnitus**	*Tinnitus presence*	Tinnitus presence or absence	ElBeltagy et al. (2012), Saliba et al. (2015)
		Tinnitus prevalence	Sarafraz et al. (2015)
		3-point likert scale: absent (tinnitus disappeared), intermittent (symptoms appeared and disappeared intermittently), continuous (symptoms were always present throughout the day)	Morales-Luckie et al. (2005)
	Tinnitus frequency	Tinnitus frequency (unspecified)	Lambert et al. (2016), Martini et al. (1990), Schmidt et al. (1992)
		Tinnitus (unspecified)	Teggi et al. (2008), Thomsen et al. (1998)
	Tinnitus severity	10-point likert scale: visual analog scale (0-10)	Ödkvist et al. (2000), Rask-Andersen et al. (2005), Scott et al. (1994), Thomsen et al. (2005)
		10-point likert scale: mild = rated on VAS 1 – 3, moderate = rated on VAS 4 – 7 severe = rated on VAS 8 – 10	Khan et al. (2011)
		7-point likert scale: 0= absent, 1 = rarely present, 2 = occasionally present, 3 = frequently but fluctuating in intensity, 4 = constantly but tolerable, 5 = constantly and troubling, 6 = very intense	Salami et al. (1984), Ricci et al. (1987)
		5-point rating scale: (intensity) 0 = absent, 5 = disabling	Martini et al. (1990)
		4-point likert scale: 0 = not present, 1 = present but moderate, 2 = severe but not incapacitating 3 = incapacitating	Elia et al. (1964)

		4-point likert scale: 0 = none; 1 = noticeable in quiet environments; 2 = occasionally noticeable, 3 = noticeable throughout the entire day	Miura et al. (1994)
		4-point likert scale: 0 = none, 1 = mild, 2 = moderate, 4 = severe	Kitahara (1986), Postema et al. (2008), Moser et al. (1984), Schmidt (1992)
		4-point likert scale: 0 = none, 1 = slight, 2 = moderate, 4 = severe	van Deelen et al. (1986)
		4-point likert scale (unspecified)	Frew et al. (1976), Wilmot et al. (1976)
		3 point likert scale: 0 = none, 1 = little bit, 2 = extreme	Okamoto et al. (1968)
		3-point likert scale (unspecified)	Thomsen et al. (1981)
		Tinnitus visual scale (unspecified)	Densert et al. (1997)
		Tinnitus sound matching contralateral ear	Rask-Andersen et al. (2005), Scott et al. (1994), Yang et al. (2024)
		Degree of Tinnitus loudness (unspecified)	Lambert et al. (2016), Schmidt et al. (1992), Yang et al. (2024)
		Tinnitus intensity in dB (not further specified)	Adrion et al. (2016)
		Unspecified	Novotný et al. (2002)
	Tinnitus improvement	3-point likert scale: better, worse, the same	Meyer et al. (1985), Silverstein et al. (1998)
		Improvement (unspecified)	Paragache et al. (2005)
		Unspecified	Martini et al. (1990)
**Aural fullness**	*Aural fullness presence*	Aural fullness recorded in a diary (unspecified)	Adrion et al. (2016)
		Aural fullness presence or absence	ElBeltagy et al. (2012), Saliba et al. (2015)
	Aural fullness severity	10-point likert scale: aural Fullness Scale (AFS) 0-10 analogue scale	Ödkvist et al. (2000), Patel et al. (2016), Thomsen et al. (2005), Wu et al. (2018), Wu et al. (2023)
		7-point likert scale: Fullness in ear: 0 = none, 1 = rare, 2 = occasionally, 3= frequently but without causing inconvenience, 4 = constantly but without causing inconvenience, 5 = constantly and troubling, 6 = important, impairing life and normal relationship	Salami et al. (1984), Ricci et al. (1987)
		5-point Likert scale: 0 =no aural fullness, 5 = the worst possible status	Morales-Luckie et al. (2005)
		4-point likert scale: 3 = severe, 2 = moderate, 1 = mild, 0 = none	Kitahara (1986), Postema et al. (2008), Moser et al. (1984), Schmidt et al. (1992)
		4-point likert scale (unspecified)	Frew et al. (1976), Wilmot et al. (1976)
		3-point likert scale (unspecified)	Thomsen et al. (1981)
		Vertigo visual scale (unspecified)	Densert et al. (1997)
		Unspecified	Novotný et al. (2002)
	Aural fullness improvement	10-point likert scale: subjective improvement scale (0-10) 0 = no changes, 10 = 100% improvement	Garduño Anaya et al. (2005)
		3-point likert scale: better, worse, the same	Meyer et al. (1985), Silverstein et al. (1998)
**Quality of life**	*Quality of life related to dizziness*	Dizziness Handicap Inventory (DHI)	Alex et al. (2024), Bremer et al. (2014), Bojrab et al. (2018), Covelli et al. (2017), Garcia et al. (2013), Garduño Anaya et al. (2005), Derebery et al. (2004), Guyot et al. (2008), Liu et al. (2020), Mira et al. (2003), Patel et al. (2016), Park et

		al. (2016), Rizk et al. (2024), Storper et al. (1998), Thomas et al. (2021), Wu et al. (2018), Wu et al. (2023), Yang et al. (2024),Yin et al. (2022), Zarandi et al. (2023), Zhuang, Y et al. (2022)
	Functional Level Score (FLS, AAO-HNS 1995)	Albu et al (2015), Alex et al. (2024), Bojrab et al. (2018), Derebery et al. (2004), Russo et al. (2016), Casani et al (2011), Patel et al. (2016), Park et al. (2016), Yardley et al. (2006), Covelli et al. (2017), Garduño Anaya et al. (2005), Thomas et al. (2021), Thomsen et al. (2005)
	European Evaluation of Vertigo scale (EEV)	Redon et al. (2013)
	Vertigo Symptom Scale short form (VSS-SF)	Patel et al. (2016), Yardley et al. (2006)
	Menière's Disease Patient-Oriented Symptom Severity Index (MD-POSI)	Lambert et al (2012), Rizk et al. (2024)
	Dizziness Beliefs Questionnaire (DBQ)	Yardley et al. (2006)
	Vestibular Disorders Activities of Daily Living (VDADL) score	Adrion et al. (2016)
	Menière's Disease Outcomes Questionnaire (MDOQ)	Bojrab et al. (2018)
	Neuropsychological Vertigo Inventory (NVI)	Rizk et al. (2024)
Quality of life related to tinnitus	Tinnitus Handicap Inventory (THI-25)	Albu et al. (2015), Alex et al. (2024), Bojrab et al. (2018), Derebery et al. (2004) Garduño Anaya et al. (2005), Lambert et al. (2012), Lambert et al. (2016), Patel et al. (2016), Park et al. (2016), Storper et al. (1998), Thomas et al. (2021), Wu et al. (2018),Wu et al. (2023), Yang et al. (2024), Yin et al. (2022), Zarandi et al. (2023)
	Mini tinnitus impairment questionnaire (MiniTF12)	Anrion et al. (2016)
Quality of life related to general health	Short form-36 (SF-36)	Derebery et al. (2004), Guyot et al. (2008), Lambert et al. (2016), Phillips et al. (2023), Wu et al. (2023)
	20-Item Short Form Health Survey (SF-20)	Rizk et al. (2024)
	Modified Somatic Perception Questionnaire (MSP)	Storper et al. (1998)
	Single questionnaire: did you feel: better, much the same, or worse than when they first completed the questionnaire?	Yardley et al. (2006)
	5-point rating scale; (Impairment of fitness for work) 5 = very much improved, 4 = much improved, 3 = slightly improved, 2 = not improved and 1 = deteriorated	Novotný et al. (2002)
	Functionality profile (unspecified)	Ödkvist et al. (2000
Quality of life related to psychological health	Hospital Anxiety and Depression Scale (HADS)	Yardley et al. (2006)
	Self-rating Depression Scale (SDS)	Kitahara et al. (2016)
	Beck Depression Scale (BDS)	Storper et al. (1998)
	Stress response scale (SRS-18)	Kitahara et al. (2016)

		Columbia suicide rating scale (C-SSRS)	Lambert et al. (2016)
		Penn State Worry Questionnaire (PSWQ)	Rizk et al. (2024)
**Other**	*Vestibular function*	Electronystagmography - Caloric test	Adrion et al. (2016), Bae et al. (2021), ElBeltagy et al. (2012), Garduño Anaya et al. (2005), Gürkov et al. (2012), Kitahara et al. (2008), Meyer et al. (1985), Moser et al. (1984), Novotný et al. (2002), Martini et al. (1990), Salami et al. (1984), Saliba et al. (2015), Scott et al. (1994), Silverstein et al. (1998), Stokroos et al. (2004), Storper et al. (1998), Schmidt et al. (1992), Wilmot et al. (1976), Wu et al. (2023)
		Video Head Impulse test (vHIT)	Wu et al. (2023)
		Cervical Vestibular Evoked Myogenic Potential (cVEMP)	ElBeltagy et al. (2012)
		Vestibulospinal test (craniocorpography)	Novotný et al. (2002)
		Rotary chair	ElBeltagy et al. (2012), Schmidt (1992), Wilmot et al. (1976)
	Auditory function	Electrocochleography	Covelli et al. (2017), Densert et al. (1997), Gates et al. (2004), Garduño Anaya et al. (2005), Ödkvist et al. (2000), Paragache et al. (2005), Park et al. (2016), Schmidt et al. (1992), Yang et al. (2024)
	Balance	Balance Rehabilitation Unit (BRU)	Garcia et al. (2013)
		Changes in upright balance = the mean and maximum trajectory excursion in the mediolateral and anteroposterior directions and a statokinesigram	Hsu et al. (2015)
		Postural control, oculomotor control, verticality perception in subjective visual vertical	Redon et al. (2013)
		The Tetrax Fall index	Liu et al. (2020)
		Clinical balance tests: positional, optokinetic	Wilmot et al. (1976)
		Unterberger's test-linear displacement, lateral sway, angular deviation, body spin; Romberg's test anteroposterior and lateral sway	Novotný et al. (2002)
		5-point likert scale: gait disturbance: 1 = very good improvement of symptoms (free of symptoms), 2 = good improvement (significant reduction of symptom severity), 3 = slight improvement (noticeable reduction in symptoms), 4 = no improvement 5 = increase in symptoms	Meyer et al. (1985)
		3-point likert scale: imbalance (standing man) 0 = none, 1 = little bit, 2 = extreme	Okamoto et al. (1968)
		Spontaneous vestibular signs: gait (normal/altered), romberg test (normal/altered), spontaneous nystagmus (present/absent)	Salami et al. (1984)
	Cognitive function	Cognitive Failure Questionnaire (CFQ)	Rizk et al. (2024)
	Compliance to treatment	Telephone calls + return empty medication packages	Albu et al. (2015)
	Tolerability to treatment	Problematic Experience of Therapy Scale (PETS)	Yardley et al. (2006)

Improvement after treatment	5-point rating scale: 5 = very much improved, 4 = much improved, 3 = slightly improved, 2 = not improved and 1 = deteriorated	Novotný et al. (2002)
	5-point likert scale: significant improvement, moderate improvement, slight improvement, no change, and deterioration	Miura et al. (1994)
	5-point likert scale: I = significant improvement, II = moderate improvement, III = mild improvement, IV = no change, V = Worsening	Kitahara et al. (1986)
	5-point likert scale (unspecified)	Mira et al. (2003)
	5-point likert scale: extremely useful, very useful, somewhat useful, not useful, and unfavorable	Miura et al. (1994)
	5-point likert scale: I = Extremely useful, II = Useful, III = Slightly useful, IV = Indeterminate, V = Not useful	Kitahara et al. (1986)
	3-point likert scale: global assessment of vertiginous pathology = improved, stable, worsened	Martini et al. (1990)
	Patient Global Impression of Change (PGIC)	Phillips et al. (2023)
	Overall effect	Thomsen et al. (1981)
Quality of appointments	Patient Enablement Instrument (PEI)	Yardley et al. (2006)
Laboratory tests	Stress and psychological factors: laboratory tests; stress-related molecules, plasma vasopressin, serum cortisol	Kitahara et al. (2016)
	Systemic dexamethasone levels	Lambert et al (2012)
	Blood pressure, blood routine, liver and kidney functions, blood fat, ECG	Guo Fang. (2007)
	Blood cell count, serum creatinine, serum glucose, AST/ SGOT, ALT/SGPT, γ-GT, alkaline phosphatase, BUN, serum bilirubin, serum sodium, serum potassium and urine analysis	Mira et al. (2003)
	Glucose, urea, creatinine, and electrolytes	Morales-Luckie et al (2005)
	Blood pressure, serum glycerol, furosemide test, potassium, sodium chloride and glucose	van Deelen et al. (1986)
	Red blood cell count, white blood cell count, hemoglobin level, GOT, GPT, ALP, BUN, Na, K, Ca, Cl, LDH	Kitahara et al. (1986)
	Vital signs and laboratory results (not further specified)	Adrion et al. (2016), Lambert et al. (2012), Lambert et al. (2016)
Activity level	5-point likert scale: activity level scale: 0 = no reduction in activity, 1 and 2 = minor or moderate reductions in activity, respectively, without having to cancel a planned schedule, 3 = need to stay at home, leave work or cancel a planned schedule, 4 = bedridden or largely incapacitated during that day.	Gates et al. (2004), Gürkov et al. (2012)
Symptoms of Menière's Disease	Gibson score	Hanner et al. (2010)
Severity of the disease	10-point likert scale (unspecified)	Thomsen et al. (1981)
Nausea/vomiting	4-point likert scale: 0 = absent, 1 = mild, 2= severe, 3 = very severe	Moser et al. (1984)
	4-point likert scale 0 = not present, 1 = present but moderate, 2 = severe but not incapacitating 3 = incapacitating	Elia et al. (1964)
	4-point likert scale: 0 = absent, 1 = mild, 2 = moderate, 3 = severe	Kitahara et al. (1986)
	4-point likert scale (severity)	Wilmot et al. (1976)
	3-point likert scale (unspecified)	Thomsen et al. (1981)
	Unspecified	Salami et al. (1984), Novotný et al. (2002)

Headache	4-point likert scale: 0 = absent, 1 = mild, 2= severe, 3 = very severe	Moser et al. (1984)
	4-point likert scale: 0 = not present, 1 = present but moderate, 2 = severe but not incapacitating 3 = incapacitating	Elia et al. (1964)
	4-point likert scale: 0 = absent, 1 = mild, 2 = moderate, 3 = severe	Kitahara et al. (1986)
	Unspecified	Novotný et al. (2002)
Otalgia	4-point likert scale: 0 = not present, 1 = present but moderate, 2 = severe but not incapacitating 3 = incapacitating	Elia et al. (1964)
Nystagmus	4-point likert scale: 0 = not present, 1 = present but moderate, 2 = severe but not incapacitating 3 = incapacitating	Elia et al. (1964)
Vegetative symptoms	5 point rating scale 0 = absent, 5 = disabling	Martini et al. (1990)
	Unspecified	Novotný et al. (2002)
Stiffness in shoulders and neck	4-point likert scale: 0 = absent, 1 = mild, 2 = moderate, 3 = severe	Kitahara et al. (1986)
